# The “social brain” is highly sensitive to the mere presence of social information: An automated meta-analysis and an independent study

**DOI:** 10.1371/journal.pone.0196503

**Published:** 2018-05-03

**Authors:** Ivy F. Tso, Saige Rutherford, Yu Fang, Mike Angstadt, Stephan F. Taylor

**Affiliations:** 1 Department of Psychiatry, University of Michigan Medical School, Ann Arbor, Michigan, United States of America; 2 Department of Psychology, University of Michigan, Ann Arbor, Michigan, United States of America; University of Texas at Austin, UNITED STATES

## Abstract

How the human brain processes social information is an increasingly researched topic in psychology and neuroscience, advancing our understanding of basic human cognition and psychopathologies. Neuroimaging studies typically seek to isolate one specific aspect of social cognition when trying to map its neural substrates. It is unclear if brain activation elicited by different social cognitive processes and task instructions are also spontaneously elicited by general social information. In this study, we investigated whether these brain regions are evoked by the mere presence of social information using an automated meta-analysis and confirmatory data from an independent study of simple appraisal of social vs. non-social images. Results of 1,000 published fMRI studies containing the keyword of “social” were subject to an automated meta-analysis (http://neurosynth.org). To confirm that significant brain regions in the meta-analysis were driven by a social effect, these brain regions were used as regions of interest (ROIs) to extract and compare BOLD fMRI signals of social vs. non-social conditions in the independent study. The NeuroSynth results indicated that the dorsal and ventral medial prefrontal cortex, posterior cingulate cortex, bilateral amygdala, bilateral occipito-temporal junction, right fusiform gyrus, bilateral temporal pole, and right inferior frontal gyrus are commonly engaged in studies with a prominent social element. The social–non-social contrast in the independent study showed a strong resemblance to the NeuroSynth map. ROI analyses revealed that a social effect was credible in 9 out of the 11 NeuroSynth regions in the independent dataset. The findings support the conclusion that the “social brain” is highly sensitive to the mere presence of social information.

## Introduction

Social cognition—the cognitive processes involved in processing information about self, other people, interpersonal relationships, and social interactions [1]—is critical to social development and adaptation. Understanding how the human brain processes social information has been an increasingly important topic in psychology and neuroscience. Research in this area not only has increased our knowledge of the functional specialization and organization of the healthy brain, but also provides a promising avenue to uncover the pathogenesis of complex neuropsychiatric disorders in which abnormal social information processing is a prominent feature, such as schizophrenia [2] and autism [3].

Social cognition comprises many cognitive processes, including perception of socially relevant cues (faces, eye gaze, facial expressions, prosody, body movements and gesture), understanding and making inferences about others' mental state, forming judgments of others, and reflection on the self and its relation to others. Several brain regions are often implicated in social information processing, including the medial prefrontal cortex (mPFC), superior temporal sulcus/gyrus (STS/STG), fusiform gyrus, temporo-parietal junction (TPJ), temporal pole, precuneus/posterior cingulate cortex (PCC), and amygdala [4–6]. The wealth of research data in this area has provided some clues about the roles of these brain regions in social information processing, although delineating their fine-grained functional specializations is still an active topic of investigation. Some conclusions about specialized social functions have emerged from the literature. For example, the mPFC appears to be associated with forming meta-representations of the self and the mental states of other people [7]; lesions in this region result in deficits in interpreting nonverbal social information, recognizing social faux pas and sarcasm, and showing empathic concern for others [8–11]. Activity in the posterior STS/STG, fusiform gyrus, and anterior temporal cortex is elicited by face and eye gaze processing [12,13], observation of biological motion [14], and inferring intentions from others’ actions [15,16]. Activity in the TPJ is associated with both mental and spatial perspective taking [17,18] and understanding false beliefs [19,20]. The PCC is thought to integrate emotional and autobiographical memory in the personal context during self-referential information processing [21]. The amygdala is involved in making judgments about faces and shows increased activation to untrustworthy relative to trustworthy faces [22].

In order to map neural substrates of social cognition, neuroimaging researchers typically design a task which seeks to isolate one specific process, such as theory of mind [23], leading to activation patterns thought to be specific to that process. In this study, we took a slightly different approach, namely to address the question as to *whether these brain regions are evoked by the mere presence of social information*. One possibility is that very specific tasks are required to activate these regions; whereas the alternative possibility is that the mere presence of social information, regardless of task instructions, is sufficient to activate these areas. One might expect the latter case, given that social processing is so robust and deeply programmed into the cognitive-perceptual machinery that they are easily ‘turned on.’ To test the hypothesis that most of the regions described above are more generally dedicated to social information, we employed a two-step strategy. Using an automated meta-analysis, NeuroSynth (neurosynth.org; 30), we first identified the brain regions found in published neuroimaging studies that contained the word ‘social’ in the text at a prominent frequency (> 1 in 1000 words), regardless of specific tasks, instructions, and contrasts. This affords us an inclusive and comprehensive picture of the “social brain regions” commonly appearing in the literature, but elicited by a variety of tasks and paradigms (e.g., facial emotion discrimination, social and moral judgment, theory of mind, social exclusion, gambling task, rewarding processing, response inhibition) and stimuli (e.g., faces, geometric shapes, cartoon strips, vocal sounds, speech). In a second step, we conducted an independent neuroimaging study in which subjects viewed complex visual images depicting social and non-social scenes of varying emotional valence. Subjects performed a simple valence appraisal task in which the socialness of the stimuli was manipulated while holding constant other stimuli characteristics (e.g., visual complexity, valence, arousal). Although this task involved specific cognitive processes related to valence appraisal, by contrasting the social and non-social conditions, we were able to isolate brain activation specific to the mere presence of information that is social in nature. We hypothesized that a majority of the brain regions identified using the NeuroSynth results would show preferential activation for social (as opposed to non-social) images in the independent study, providing support for the hypothesis that “the social brain” is very sensitive to the mere presence of social information.

## Materials and methods

### Automated meta-analysis

The first part of the analyses of this study aimed to identify the brain regions that have shown significant activation in published fMRI studies with a prominent social element in the literature. Using the keyword “social” yielded 1,000 published fMRI studies to include in an automated meta-analysis on neurosynth.org (http://neurosynth.org/analyses/terms/social/) [24]. We used the reverse inference map of the result of the automated meta-analysis, which represents z-scores corresponding to the likelihood that the term “social” is used in a study given the presence of reported activation (i.e., P[Social|Activation]). It is obtained by comparing all the studies in the Neurosynth database that contain “social” and those that do not. The significant brain regions showing up in the reverse inference map represent those that are more likely to be reported in “social” studies than in “non-social” studies. In contrast, the forward inference map (P[Activation|Social]) does not consider the base-rate activation of the regions, and the result may very well include regions that are involved in almost every task. As such, the reverse inference map is a better indicator of *how specific* the activated regions are to social information processing. The activation map was thresholded at FDR-corrected *p* < 0.01 by default, yielding 135 significant clusters. The majority of the clusters were very small in terms of voxel size (< 10 voxels). Eleven clusters were ≥ 100 voxels in size and were selected to represent “social brain regions in the literature.” Since the resolution of the NeuroSynth map was higher than that of the independent dataset, the NeuroSynth results were downsampled from a voxel size of 2 × 2 × 2 mm^3^ to 3 × 3 × 3 mm^3^ to facilitate later comparisons. Masks derived from the 11 regions served as the regions of interest (ROIs) for beta extraction for the independent study.

### Independent study

#### Participants

Fifteen healthy participants were recruited from community advertisements and completed the study. All participants were free of Axis I psychiatric disorders as established with the Structured Clinical Interview for Diagnosis, non-patient version (SCID-NP) [25] and were not taking any medications. The risks of the study were explained to all participants prior to obtaining their written, informed consent to participate. The study was conducted in accordance to the study protocol with ethical standards in line with the Declaration of Helsinki and approved by the University of Michigan Medical School Institutional Review Board (IRBMED), IRBMED# 2001–0283. One participant’s fMRI data were lost due to archival errors; data of the remaining 14 (4 female) participants, aged from 23 to 50 years (mean = 38.6, SD = 10.1), were included in the analyses of this report. A previous peer-reviewed publication reported on different aspects of this sample using this paradigm [26].

#### Visual stimuli

**Fig 1:**
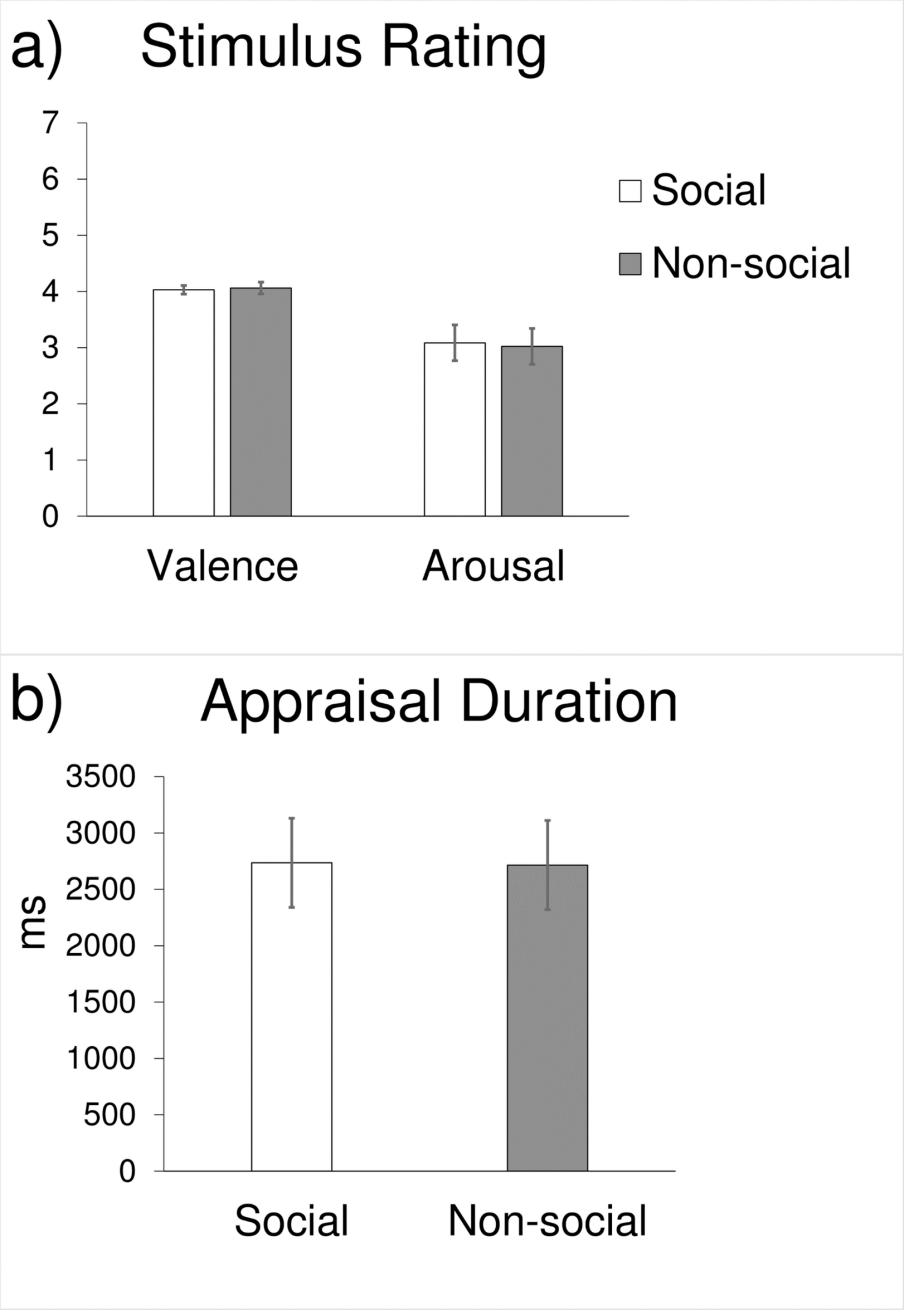
Characteristics of the social and non-social images used in this study. **a)** Subjective valance (*p* = .647) and arousal ratings (*p* = .464) by the participants did not differ significantly between the social and non-social conditions. **b)** Image appraisal time in the scanner did not differ significantly between the social and non-social conditions (*p* = .856). Vertical lines represent standard errors of mean.

One hundred and twenty (including 60 social and 60 non-social) complex images were selected from the International Affective Picture System (IAPS) [27]. Image selection began with identifying images that contained the presence of human and/or interactions between social animals as candidate social images, and those that contained landscapes or physical objects only as candidate non-social images. Candidate images were then classified as negative, neutral, and positive based on their normative valence ratings. Finally, 20 negative, 20 neutral, and 20 positive images were selected for each of the social and non-social conditions such that the two conditions were matched on valence and arousal based on the normative ratings associated with each image. Some examples of these images included: a gory face (social, negative), a soiled toilet (non-social, negative), a man facing a computer monitor (social, neutral), a bus (nonsocial, neutral), two children playing with cats (social, positive), and a colorful flower field (nonsocial, positive). Equivalent valence and arousal of the social and non-social images was later confirmed using the subjective ratings by the participants in the independent study (see Fig 1A and more details of procedure below). A complete list of the IAPS images used in the independent study can be found in supporting information S1 Table.

In addition to the IAPS images, “blank” (BL) images were included as a baseline condition. They were 4 unique kinds of images composed of a colored polygon against a lightly textured, gray-toned background of varying shades. The contrast and brightness of each set of the images were adjusted to match on total luminance using Photoshop 4.0 (Adobe Systems).

#### Appraisal task: Design and procedure

The images were presented in 20-second blocks; each block consisted of 4 images and each image was presented for 5 seconds. For each image, participants were instructed to form a judgment as to whether it was pleasant, neutral, or unpleasant, and to press a button to signal that they had formed a judgment. Appraisal duration for social and non-social images did not differ significantly (Fig 1B). The task consisted of a total of 30 blocks of IAPS images divided into 5 runs. Blocks of IAPS images alternated with blocks of BL images. Participants completed a practice session before the fMRI scanning to ensure comprehension of the task. Participants’ attention was monitored using an eye tracker in the scanner.

Immediately after the fMRI session, participants viewed all of the IAPS images again on a computer outside of the scanner, presented in a randomized order, and rated each image for valence and arousal on a 7-point scale. For valence, the prompt question was “How pleasant or unpleasant does this picture make you feel?” and participants chose a number between 1 and 7, with 1 = “Extremely unpleasant,” 2 = “Very unpleasant,” 3 = “Mildly unpleasant,” 4 = “Neither,” 5 = “Mildly pleasant,” 6 = “Very pleasant,” and 7 = “Extremely pleasant.” For arousal, the prompt question was “How calm or excited/aroused does this picture make you feel?” and participants chose a number between 1 and 7, which was accompanied by a description “Calm, not aroused/excited → A little → Moderately → Very → Extremely aroused/excited.” Social and non-social images were similar in both valence and arousal ratings, Fig 1A.

#### fMRI acquisition and processing

MRI scanning occurred on a GE 3T Signa scanner (LX [8.3] release, General Electric Healthcare, Buckinghamshire, United Kingdom). A T1-weighted image was acquired in the same prescription as the functional images to facilitate co-registration. Functional images were acquired with a T2*-weighted, reverse spiral acquisition sequence (gradient recalled echo, TR = 2000 ms, TE = 30 ms, FA = 90 degrees, field of view = 20 cm, 40 slice, 3.0mm thick/0mm skip, equivalent to 64 x 64 voxel grid) sensitive to signal in ventral medial frontal regions [28]. Subjects underwent 5 runs (6 blocks/runs), each consisting of 120 volumes, plus 4 initial, discarded volumes to allow for equilibration of scanner signal, with isotropic voxels 3 mm after normalization. After acquisition of functional volumes, a high resolution T1 scan (3D SPGR, field of view = 24 cm, TR = 25 ms, TE = 3 ms, 256 × 160 matrix, 100 slices, 1.5 mm interleaved with no skip) was obtained for anatomic normalization.

fMRI data were preprocessed with the Statistical Parametric Mapping (SPM8) package (Wellcome Institute of Cognitive Neurology, London) and FSL (FMRIB, Oxford, UK) and standard routines. Slice time was corrected using sinc-interpolation, weighted by a Hanning kernel in time. Then all scans were realigned to the 10th volume acquired during each scan ("mcflirt") [29]. Runs with movement exceeding either 1 voxel or 2 degrees rotation within a scan were discarded; only 1 run of 1 subject was discarded as a result. The time series of functional volumes were then co-registered with the high resolution T1 image, spatially normalized to the MNI152 brain, and then spatially smoothed with a 6 mm isotropic Gaussian kernel.

#### Statistical analyses

fMRI data analyses were performed with SPM12. First-level analysis began with applying a high pass filter (128 s) to the anatomically normalized time series, and regressed on 2 regressors (social, non-social) convolved with a canonical hemodynamic response function, along with 24 motion regressors (6 for each translation/rotation direction, their first derivative, and quadratic terms for each direction and derivative). BL blocks were modeled as implicit baseline.

Second-level analyses involved both whole-brain and ROI analyses. The former informs the brain regions preferentially responding to social vs. non-social stimuli; the latter reveals the extent to which “social brain regions” seen in the literature are engaged in processing the social nature of stimuli.

For the whole-brain analysis, the *t* statistics map of the social–nonsocial contrast was examined. Initial clusters were defined by a voxel threshold of uncorrected *p* < .005; “significant” clusters were determined by a threshold of false discovery error (FDR) corrected *p* < .05 based on the Gaussian random field theory [30]. Subsequently, a conjunction analysis was performed to show the overlap between social networks identified in the NeuroSynth result and our data, by first binarizing supra-threshold voxels of the NeuroSynth map and the social–nonsocial contrast map of our data, and then finding the voxels that were above threshold in both maps.

The ROI analyses involved extracting beta estimates of the social and non-social conditions in the independent study from the 11 NeuroSynth-informed ROIs. This was done by saving each of the Neurosynth ROIs into separate masks, and then applying the masks to the first-level results of the social (vs. baseline) and non-social (vs. baseline) contrasts in the independent study. The first eigenvector of beta estimates from these ROIs was extracted and subject to Bayesian inference. Specifically, we used the anovaBF command of the R package “BayesFactor” [31] to compare evidence of two competing models—a model containing Socialness as a fixed factor and a null model—for each brain region given the data; subjects were modeled as a random factor in both models. Relative evidence strength of the two models was expressed in Bayes factor, such that a value < 1 indicates evidence favoring the denominator model (null model) over the numerator model (Social effect model), whereas a value > 1 indicates evidence favoring the numerator model over the denominator model. Further, interpretation of *strength of evidence* followed guidelines by Jeffreys [32], where Bayes factors between 3 and 10 indicate that the support for the Social effect model is “substantial,” values between 10 and 30 “strong,” values between 30 and 100 “very strong,” and values > 100 “decisive”; similarly, values between 0.10 and 0.33 indicate “substantial” support for the null model, values between 0.033 and 0.10 “strong,” values between 0.01 and 0.033 “strong” support, and values < 0.01 “decisive.”

## Results

### Whole-brain analyses

From the NeuroSynth data, 11 clusters were identified as “social” brain regions in the literature: dorsomedial PFC (dmPFC), ventromedial PFC (vmPFC), PCC, bilateral amygdala, right fusiform gyrus, bilateral OTJ, bilateral anterior temporal cortex/temporal pole, and right inferior frontal gyrus (IFG) (Table 1). Please note that results are in reduced resolution of voxel size 3 × 3 × 3 mm^3^ for easier comparison with the results of the independent dataset in Table 2.

**Table 1:** “Social” brain regions identified in the NeuroSynth meta-analysis.

Area	Peak Z	Cluster size	Center-of-mass coordinate (x, y, z)
Dorsomedial prefrontal cortex (dmPFC)	9.25	494	-1.6, 54.2, 27.6
Ventromedial prefrontal cortex (vmPFC)	7.04	193	1.9, 47.2, -14.9
R occipito-temporal junction (OTJ)	7.26	288	52.3, -51.0, 12.5
L occipito-temporal junction (OTJ)	6.91	177	-52.8, -60.0, 18.6
Precuneus / posterior cingulate cortex (PCC)	9.25	165	-0.7, -55.5, 34.0
R temporal pole	7.24	209	52.0, 3.4, -27.4
L temporal pole	6.50	195	-45.5, 13.1, -23.6
R inferior frontal gyrus (IFG) / orbito-frontal cortex (OFC)	8.13	107	49.8, 28.7, -2.1
R amygdala	7.82	103	19.9, -2.0, -18.8
L amygdala	6.82	74	-20.8, -4.7, -18.0
R fusiform gyrus	6.11	69	43.2, -44.5, -21.4

L = left; R = right. Clusters were significant at FDR-corrected *p* < 0.01.

**Table 2:** Brain regions showing increased BOLD signals during processing of social vs. non-social images.

Area	Cluster (voxels)	Peak Z	x, y, z
Medial prefrontal cortex (mPFC) extending across ventral and dorsal regions	210	4.01	-6, 56, -14
	a	3.61	-6, 56, 16
	a	3.24	0, 53, -8
	a	4.64	-36, -7, -20
R occipito-temporal junction (OTJ)	695	5.14	48, -73, 4
	a	4.65	54, -40, 16
	a	4.45	51, -79, -5
L occipito-temporal junction (OTJ) / fusiform gyrus	1006	5.06	-54, -73, 7
	a	4.65	-36, -58, -17
	a	4.47	-45, -82, -2
Precuneus / posterior cingulate cortex (PCC)	704	4.67	-3, -46, 22
	a	4.64	3, -61, 25
	a	4.62	-3, -58, 49
R amygdala / hippocampus / temporal pole	144	3.85	33, -1, -20
	a	3.62	54, -10, -26
	a	3.57	45, -4, -23
R superior parietal cortex	66	3.85	30, -43, 67
	a	2.92	27, -49, 55
R fusiform gyrus	91	3.75	42, -61, -20
	a	3.60	42, -46, -23
	a	3.18	45, -37, -20
L superior / middle frontal gyrus	84	4.04	-18, 32, 40
	a	3.73	-21, 23, 46
	a	3.31	-12, 29, 46

BOLD = blood oxygenation level-dependent. L = left; R = right. All clusters were significant at FDR-corrected *p* < 0.05. a. Peak voxel part of a single super-cluster.

Results of the social–nonsocial contrast of our data revealed that social images, compared with nonsocial images, elicited significantly higher activation in a number of brain areas, including mPFC extending across ventral and dorsal areas, PCC, bilateral amygdala extending to hippocampus and anterior temporal cortex/temporal pole, bilateral OTJ extending to fusiform gyri, right superior parietal cortex, and left superior/middle frontal gyrus (Table 2).

**Fig 2:**
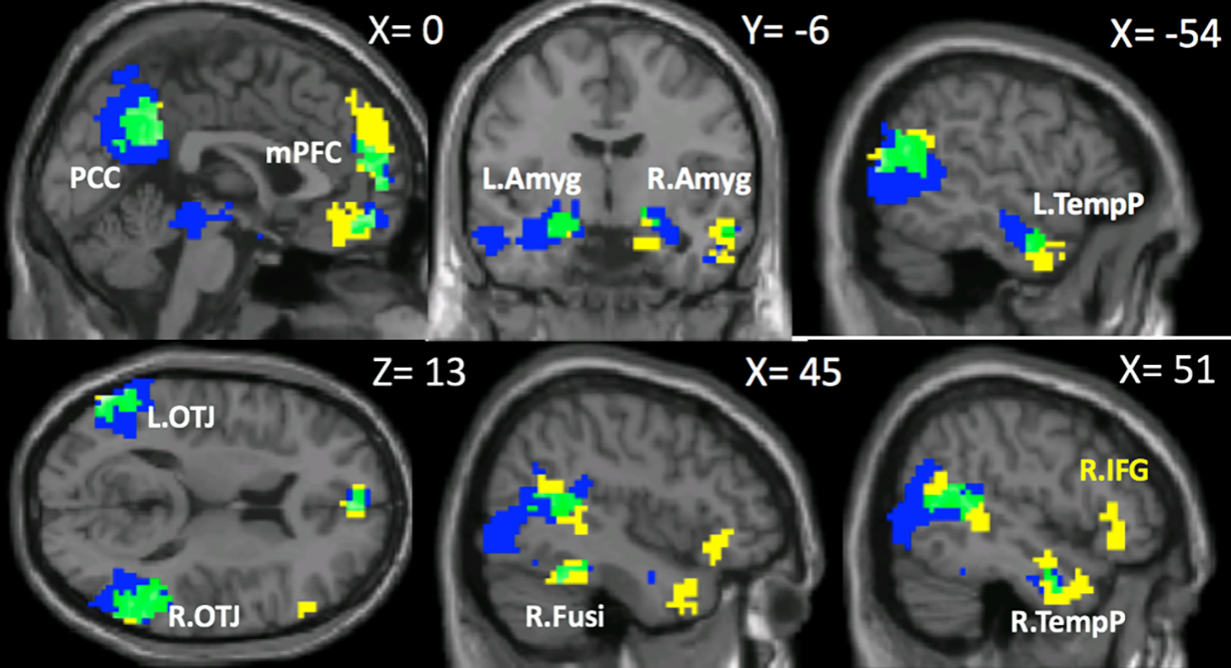
“Social” brain regions. Areas identified in the NeuroSynth meta-analysis result (yellow) and brain regions showing preferential activation to social stimuli in our data (blue) showed remarkable overlap (green). Regions significant in both our data and the NeuroSynth results are labeled in white, those significant only in NeuroSynth are labelled in yellow. mPFC = medial prefrontal cortex; PCC = posterior cingulate cortex; L.Amyg = left amygdala; R.Amgy = right amygdala; R.Fusi = right fusiform gyrus; L.OTJ = left occipito-temporal junction; R.OTJ = right occipito-temporal junction; L.TempP = left temporal pole; R.TempP = right temporal pole; R.IFG = right inferior frontal gyrus.

Overall, the social brain regions identified using the NeuroSynth data and our data showed a strong resemblance. See Fig 2 for the results of the conjunction analysis, showing simultaneously the NeuroSynth map and the social–nonsocial contrast of the independent study, as well as their overlap.

**Fig 3:**
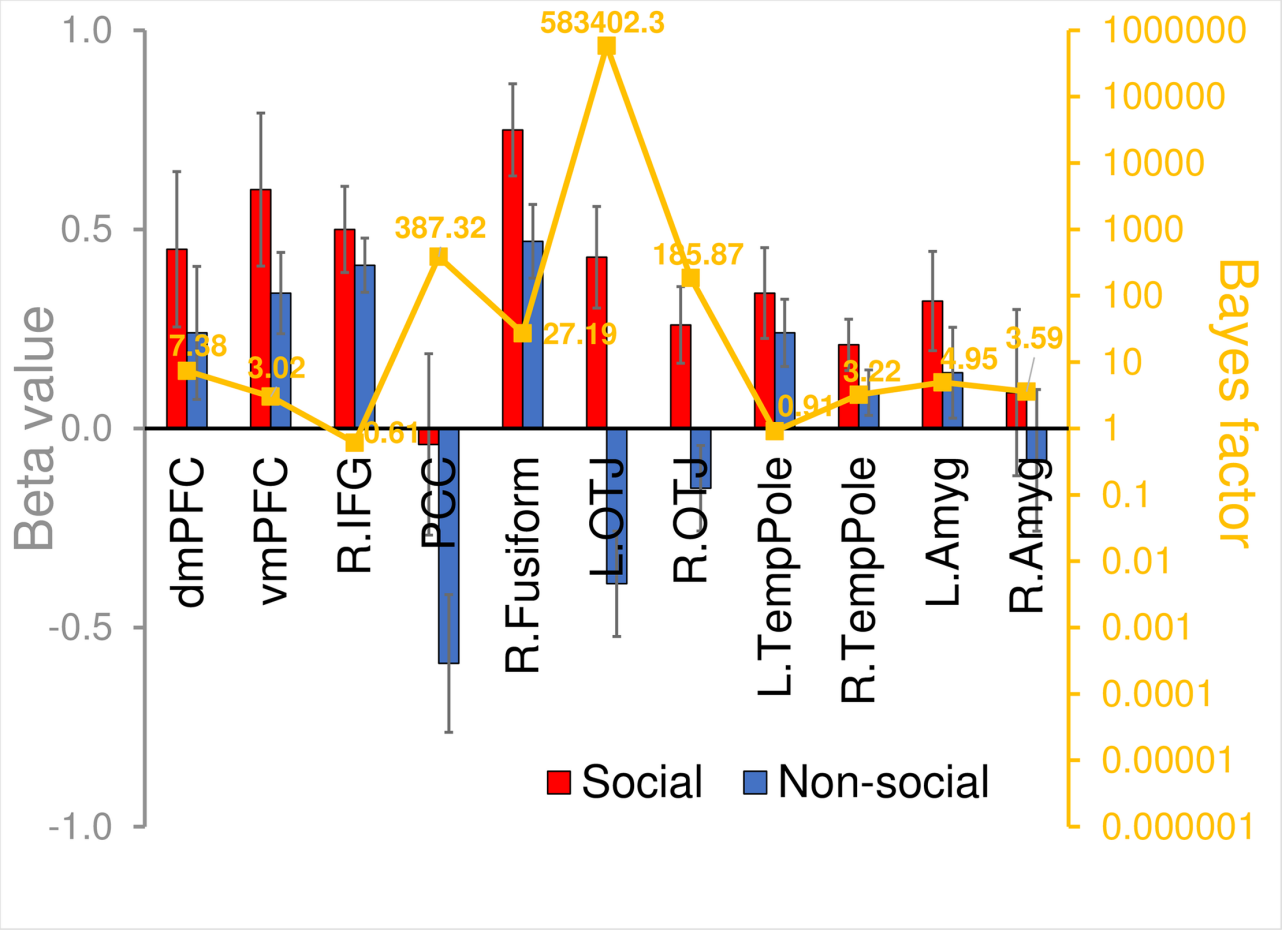
BOLD signals in social and non-social conditions in the independent dataset in the 11 NeuroSynth “social” brain regions. Bars (left y-axis) represent beta estimates and vertical lines represent standard errors of mean. The line (right y-axis) indicates Bayes factor values comparing a model with Socialness as a fixed effect (numerator) against a null model (denominator). dmPFC = dorsomedial prefrontal cortex; vmPFC = ventromedial prefrontal cortex; R.IFG = right inferior frontal gyrus; PCC = posterior cingulate cortex; R.fusiform = right fusiform gyrus; L.OTJ = left occipito-temporal junction; R.OTJ = right occipito-temporal junction; L.TempPole = left temporal pole; R.TempPole = right temporal pole; L.amyg = left amygdala; and R.amyg = right amygdala.

Beta estimates of the social and non-social conditions in our data extracted from the NeuroSynth-informed ROIs, and the results of statistical tests of a Social effect in these ROIs, are displayed in Fig 3. In all 11 ROIs, social images elicited higher activation than non-social images. Bayesian evidence favored the presence of a Social effect (Bayes factor > 1) in 9 out of the 11 regions (i.e., all but R IFG and L temporal pole). The evidence for a Social effect was “substantial” or stronger (Bayes factor > 3) in all of these 9 regions—dmPFC, vmPFC, PCC, R fusiform gyrus, bilateral OTJ, R temporal pole, and bilateral amygdala.

## Discussion

This study investigated if there are sensitive, task-general modules for processing social information in the human brain. We first examined the brain regions commonly activated in a large number (*N* = 1,000) of published fMRI studies involving a prominent social element using the automated meta-analysis method provided by NeuroSynth [24]. The NeuroSynth map revealed distributed neural substrates related to social processing, including the ventral and dorsal areas of the mPFC, precuneus/PCC, bilateral amygdala, bilateral OTJ (extending to fusiform gyrus), bilateral anterior temporal cortex/temporal pole, and inferior frontal gyrus extending to orbitofrontal cortex. This map is highly consistent with brain regions often implicated in socio-emotional processing in the literature. Then we evaluated if these brain regions are representative of social processing by conducting confirmatory analyses on an independent dataset that specifically compared the socialness of the stimuli. By carefully matching the affective valence and levels of arousal of the images used in the social and non-social conditions, we isolated social processing from other cognitive processes on brain activation. Overlaying the results of the independent study on the NeuroSynth “social” map showed a strong correspondence of the two maps. ROI analyses examining brain activation in these NeuroSynth regions in our data showed that a credible social effect (social > non-social) was present in 9 out of 11 of these regions. Taken together, the results of this study provided convincing support that a number of brain regions in the human brain are robustly and preferentially activated when processing social information.

The similarities between the NeuroSynth map and the social–non-social contrast of the independent dataset are remarkable given the differences in methods used to generate the two maps. The NeuroSynth methods elicit very crude “contrasts”–the studies included were those in which the term ‘social’ appear in the article text at a “high” frequency (defined as > 1 in every 1,000 words), and the coordinates that went into the meta-analysis were automatically extracted from all tables reported in these studies, regardless of contrasts or (sub)groups. In the independent study, participants were only given a vague task (to “form a judgment” of the pleasantness of each of the images), rather than told explicitly to attend to the social aspect of the images or to perform a specific social cognitive task. Additionally, the use of the social–nonsocial contrast theoretically canceled out common cognitive processes (particularly, valence appraisal) involved in the two conditions, making it reasonable to assume that the result reflects brain activation associated with the sociality of the stimuli only. The results showed that most of the brain regions from the NeuroSynth map were preferentially engaged in response to the mere presence of sociality in stationary scenes of humans and social interactions, consistent with the assertion that most social signals are processed nearly automatically [6]. The strong correspondence between the NeuroSynth and the independent study suggests that regardless of tasks and methods, certain cognitive processes are easily involved in processing information social in nature: analysis of postures and biological motion (OTJ) [33], accessing social knowledge (temporal pole) [34], autobiographical recollection (PCC) [35], and reflection on feelings and self-reference (mPFC) [7]. Further, the conjunction analysis showed extensive overlap between the NeuroSynth map and the whole-brain analysis of the independent dataset. Such overlapping regions may indicate subregions of the general social brain areas that are sensitive to the degree of sociality.

Some brain regions from the NeuroSynth map did not show a credible social effect in the independent data, such as the left temporal pole and right IFG. Additionally, some brain regions that are often implicated in social cognitive processes (e.g., TPJ as involved in theory of mind) did not show up in either the NeuroSynth map or our data. As noted in a review of the social brain [4], brain regions involved in social cognition are modulated by the task context and individual factors such as volitional regulation. The lack of a credible social effect in the left temporal pole and the right IFG in the independent data could be due to that cognitive processes recruiting the left temporal pole (e.g., semantic representation of sounds or objects) and the IFG (e.g., response inhibition) may be prevalent among studies included in the Neurosynth map but not required in the appraisal task in the independent study. Similarly, areas such as TPJ did not appear in both the NeuroSynth and the independent study maps may be because mental state attribution was not explicitly required in many of the studies included in the automated meta-analysis. Therefore, the brain regions revealed in our dataset and the NeuroSynth map should not be considered the complete “social circuitry” given the simple task used in this study and the variable representation of different social cognitive processes in the literature. While the findings provide strong support for social brain modules such as mPFC, PCC, right anterior temporal cortex, amygdala, and OTJ (extending to fusiform gyrus), negative findings in TPJ and other regions do not mean that they are not involved in social information processing. In a similar vein, the “social” brain regions identified in this study should not be interpreted as responsible for *solely* social information processing, as many (if not all) brain regions are involved in multiple cognitive processes.

This study is limited by the small sample size of the independent dataset. Although we used social and non-social stimuli carefully matched for valence and arousal, literature-informed ROIs, and Bayesian statistics to increase the scientific rigor and the interpretability of the results, we acknowledge that the results may be different with a larger or different sample. The small sample also precludes the exploration of other important questions such as differences between gender and diverse populations in the social brain network. Future investigations in larger and cross-cultural studies to reveal critical biological and social factors in human social cognition are warranted.

To conclude, this study provided support that core regions of the human social brain are highly sensitive to the mere presence of social information, including the medial prefrontal cortex, posterior cingulate cortex, temporal pole, and occipito-temporal junction extending to fusiform gyrus. This knowledge may help guide future developmental and psychopathology research. For example, tracking the qualitative and quantitative changes in this “automatic” social brain over developmental or illness stages would inform whether such a neural sensitivity to social information is innate, how it is associated with other important developmental milestones and functional markers, how it is influenced by environmental and social factors (e.g., poverty, abuse), and how its alterations may be responsible for the development and symptom manifestations of different psychopathologies. Further, investigations of high-resolution brain specialization as well as anatomical, functional, and effective brain connectivity will help us gain a fuller understanding of the neural mechanisms of social information processing and how the social brain network interacts with other brain systems to guide complex social behavior in normal development and in psychopathologies with prominent social deficits. Finally, the results of this study lend support to the usefulness of NeuroSynth in neuroscience research, as it provides a relatively accurate picture of the neural substrates of a variety of broadly conceived cognitive processes.

## Supporting information

S1 Table International Affective Picture System (IAPS) images used in the independent study. (DOCX)
