# Supplementary material for: The “social brain” is highly sensitive to the mere presence of social information: An automated meta-analysis and an independent study
Source: PLoS One. 2018 May 3;13(5):e0196503. doi: 10.1371/journal.pone.0196503 (PMC5933734; doi:10.1371/journal.pone.0196503)
Supplement: S1 Table — (DOCX) [file pone.0196503.s001.docx]

**S1 Table. International Affective Picture System (IAPS) Images Used in the Independent Study.**

| **Social** | | | **Non-social** | | |
| --- | --- | --- | --- | --- | --- |
| **Negative** | **Neutral** | **Positive** | **Negative** | **Neutral** | **Positive** |
| 1201 | 2190 | 1340 | 1111 | 1390 | 1560 |
| 2053 | 2200 | 1463 | 1240 | 5120 | 1650 |
| 2120 | 2271 | 1710 | 1280 | 5130 | 1900 |
| 2141 | 2372 | 1920 | 3150 | 5250 | 5220 |
| 2900 | 2383 | 2050 | 3400 | 5390 | 5260 |
| 3000 | 2440 | 2091 | 5940 | 5395 | 5270 |
| 3051 | 2487 | 2150 | 5971 | 5534 | 5300 |
| 3053 | 2516 | 2209 | 6020 | 5535 | 5594 |
| 3280 | 2560 | 2216 | 7380 | 5720 | 5600 |
| 6010 | 2575 | 2340 | 9001 | 5740 | 5626 |
| 6821 | 2580 | 2345 | 9140 | 5800 | 5660 |
| 6840 | 2840 | 2391 | 9181 | 7100 | 5760 |
| 8230 | 2850 | 2540 | 9280 | 7140 | 5780 |
| 9040 | 5410 | 4250 | 9290 | 7180 | 5849 |
| 9042 | 5875 | 4572 | 9300 | 7217 | 5910 |
| 9050 | 7550 | 4641 | 9330 | 7500 | 5990 |
| 9230 | 7620 | 5621 | 9470 | 7510 | 7230 |
| 9421 | 8160 | 8461 | 9560 | 7595 | 7270 |
| 9910 | 9070 | 8497 | 9600 | 9080 | 7330 |
| 9921 | 9210 | 8540 | 9830 | 9360 | 7570 |
